# Multi-omics derivation of a core gene signature for predicting therapeutic response and characterizing immune dysregulation in inflammatory bowel disease

**DOI:** 10.3389/fimmu.2025.1611598

**Published:** 2025-07-31

**Authors:** Mingming Wang, Liping Liang, Zibo Tang, Jimin Han, Lele Wu, Le Liu, Ye Chen

**Affiliations:** ^1^ Department of Gastroenterology, State Key Laboratory of Organ Failure Research, Guangdong Provincial Key Laboratory of Gastroenterology, Nanfang Hospital, Southern Medical University, Guangzhou, China; ^2^ Department of Gastroenterology and Hepatology, Guangzhou Key Laboratory of Digestive Diseases, Guangzhou Digestive Disease Center, Guangzhou First People’s Hospital, School of Medicine, South China University of Technology, Guangzhou, China; ^3^ Department of Radiation Oncology, Shenzhen People’s Hospital (The Second Clinical Medical College at Jinan University and The First Affiliated Hospital at the Southern University of Science and Technology), Shenzhen, China; ^4^ School of Life Sciences, Tsinghua University, Beijing, China; ^5^ Shenzhen Clinical Research Center for Digestive Disease, Integrative Microecology Clinical Center, Shenzhen Key Laboratory of Gastrointestinal Microbiota and Disease, Shenzhen Technology Research Center of Gut Microbiota Transplantation, Shenzhen Hospital, Southern Medical University, Shenzhen, China; ^6^ Department of Gastroenterology, Zhujiang Hospital, Southern Medical University, Guangzhou, China

**Keywords:** inflammatory bowel disease, machine learning, immune infiltration, biologic response prediction, precision medicine

## Abstract

**Background:**

Inflammatory bowel disease (IBD) presents unpredictable therapeutic responses and complex immune dysregulation. Current precision medicine approaches lack robust molecular tools integrating transcriptomic signatures with immune dynamics for personalized treatment guidance.

**Methods:**

We performed multi-omics analyses of GEO datasets using machine learning algorithms (LASSO/Random Forest) to derive a four-gene signature. Validation employed ten algorithms and nomogram construction. Immune infiltration (CIBERSORT/ssGSEA), single-cell RNA sequencing, and DSS-colitis models characterized immune dynamics, cellular specificity, and therapeutic response modulation.

**Results:**

We identified 536 differentially expressed genes significantly enriched in IL-17 signaling, TNF signaling, and cytokine-cytokine receptor interactions. WGCNA revealed six co-expression modules with disease-specific correlations: turquoise module strongly correlated with Crohn’s disease (r=0.6, P=4×10^-20^) and purple module with ulcerative colitis (r=0.55, P=1×10^-16^). The four-gene signature (CDC14A, PDK2, CHAD, UGT2A3) demonstrated exceptional diagnostic performance across ten validation algorithms (AUC range: 0.86-0.97), with the integrated nomogram achieving superior accuracy (AUC=0.952) compared to individual genes (CDC14A: 0.934, PDK2: 0.913, CHAD: 0.893, UGT2A3: 0.797). Consensus clustering stratified patients into two distinct molecular subtypes: Cluster 1 exhibited elevated M1 macrophages, activated dendritic cells, and neutrophils with enhanced glycolysis and mTORC1 signaling, while Cluster 2 showed higher signature gene expression, enhanced oxidative phosphorylation, and enrichment in regulatory immune populations including Tregs and M2 macrophages. Single-cell RNA sequencing revealed cell-type-specific expression patterns: PDK2 demonstrated widespread expression across epithelial cycling cells and stem cells, UGT2A3 showed preferential epithelial localization, and CDC14A exhibited selective enrichment in innate lymphoid cells. Nomogram-based risk stratification effectively predicted biologic treatment responses across multiple therapeutic classes using four independent treatment datasets (GSE16879, GSE92415, GSE73661, GSE206285): low-risk patients demonstrated superior response rates to golimumab (63.3%), infliximab (54.8%), and vedolizumab (29% vs. 15% in high-risk group). Connectivity Map analysis identified MS.275 as the top therapeutic enhancer, with experimental validation in DSS-induced colitis confirming synergistic anti-inflammatory effects with TNF-α inhibitors, improving disease activity indices and restoring signature gene expression patterns.

**Conclusion:**

This mechanistically grounded four-gene signature enables precise IBD patient stratification across distinct immunological subtypes and predicts biologic responses, providing validated molecular tools for precision immunotherapy and personalized treatment optimization.

## Introduction

Inflammatory bowel disease (IBD), encompassing ulcerative colitis (UC) and Crohn’s disease (CD), represents a chronic and relapsing inflammatory disorder primarily affecting the gastrointestinal tract. Over recent decades, the global burden of IBD has increased dramatically, particularly in industrializing nations such as China, where incidence and mortality rates sharply escalated between 1990 and 2019 (1). Although morbidity has stabilized in Western countries, the burden remains substantial with prevalence exceeding 0.3% (2). IBD imposes significant healthcare costs and profoundly impacts patients’ quality of life due to its chronic nature and associated complications.

The pathogenesis of IBD involves complex, multifactorial processes including genetic predisposition, mucosal barrier dysfunction, microbial dysbiosis, immune dysregulation, and environmental factors. Advances in genome-wide association studies (GWAS) and meta-analyses have identified numerous genetic variants associated with IBD, enhancing early diagnostic capabilities and therapeutic strategies (3). However, the precise molecular mechanisms underlying mucosal immune dysregulation remain incompletely understood. While research has traditionally implicated adaptive immune responses, such as Th1 and Th17 pathways in CD and atypical Th2 responses in UC, emerging evidence highlights the pivotal role of innate immune responses. Dysfunction in epithelial barrier integrity, microbial sensing, autophagy, and unfolded protein response are now recognized as central pathogenic mechanisms.

Despite significant advancements in pharmaceutical interventions, including aminosalicylates, corticosteroids, immunomodulators, and biologics, a considerable proportion of IBD patients fail to achieve sustained clinical remission or experience diminished therapeutic efficacy over time (4, 5). Furthermore, the long-term safety and effectiveness of emerging therapies, such as stem cell transplantation, fecal microbiota transplantation, and live biotherapeutic products, remain uncertain (6, 7). These challenges are compounded by the chronic nature of IBD and potential side effects associated with prolonged medication use.

These issues hinder the development of predictive strategies for individual IBD susceptibility and impede implementation of precise preventive measures and personalized treatment approaches. This limitation represents a significant obstacle to advancing primary prediction, targeted prevention, and personalized treatment medicine for IBD. Although the exact pathophysiological mechanisms underlying IBD remain elusive, recent research has highlighted the critical roles of core mucosal molecular characteristics and immune system dysregulation in disease etiology. Advancing our understanding of these aspects is essential for improving IBD management and developing more effective, individualized therapeutic strategies.

Recent advances in single-cell RNA sequencing (scRNA-seq) technologies have revolutionized our understanding of IBD pathogenesis by revealing unprecedented cellular heterogeneity within inflamed intestinal tissues (8). These technologies have enabled the identification of disease-specific cell states, rare cell populations, and cell-type-specific transcriptional programs that were previously masked in bulk tissue analyses. Single-cell studies have uncovered distinct epithelial cell subtypes, immune cell activation states, and stromal cell populations that contribute to IBD pathogenesis, providing crucial insights into cellular communication networks and tissue-specific responses. Concurrently, advances in bioinformatics and computational biology have facilitated sophisticated multi-omics integration approaches, enabling researchers to combine transcriptomic, epigenomic, and proteomic data to construct comprehensive molecular landscapes of IBD. Machine learning algorithms and network analysis methods have emerged as powerful tools for identifying disease-associated molecular signatures and predicting therapeutic responses (9).

Our study leverages these innovations within a comprehensive multi-omics framework, integrating differential expression analysis, weighted gene co-expression network analysis (WGCNA), machine learning, immune infiltration profiling, and drug discovery analysis. This unbiased, data-driven pipeline identified a novel four-gene signature with robust diagnostic and prognostic potential. By harnessing single-cell and bioinformatics advances, our findings offer molecular tools for risk assessment, treatment response prediction, and therapeutic decision-making, paving the way for precision medicine in IBD management.

## Materials and methods

### Data sources and preprocessing

The gene expression datasets for IBD analysis, consisting of samples from intestinal tissue, were retrieved from the Gene Expression Omnibus (GEO) database (https://www.ncbi.nlm.nih.gov/geo/). For candidate biomarker identification and validation, GSE75214 (97 UC, 75 CD, 22 healthy controls), GSE36807 (15 UC, 13 CD, 7 healthy controls), and GSE59071 (97 UC, 8 CD, 11 healthy controls) were used. To evaluate biologic treatment responses, we analyzed samples from multiple datasets: GSE16879 (28 responders and 33 non-responders to infliximab therapy at baseline), GSE92415 (32 responders and 27 non-responders to golimumab at baseline), GSE73661(6 responders and 21 non-responders to vedolizumab with matched baseline and post-treatment patient samples), and GSE206285 (49 responders and 315 non-responders to ustekinumab at baseline). For colorectal cancer (CRC) expression profile analysis, GSE39582 was selected, containing sequencing data from the GPL570 platform. Samples with available survival time, survival status, and tumor classification were retained for further analysis.

### Identification of differentially expressed genes

Differential expression analysis was performed using the GSE75214 dataset to identify genes with significantly altered expression between IBD patients and healthy controls. CD and UC patients were combined into a single “Disease” group for comparison with “Normal” controls to increase statistical power. The “limma” R package was employed for differential expression analysis using linear models with empirical Bayes moderation. DEGs were identified using criteria of p-value < 0.05 and |log2 fold change (FC)| > 1. Results were visualized using volcano plots and heatmaps generated with “ggplot2” and “pheatmap” packages, respectively.

### WGCNA

WGCNA analysis was performed using the “WGCNA” R package. Gene expression data were filtered to retain genes with standard deviation > 0.5, and sample outliers were removed using hierarchical clustering with cutoff height of 12,000. Soft threshold power = 11 was selected based on scale-free topology criteria. Gene modules were identified using dynamic tree cutting with minimum module size of 80 genes, and similar modules were merged at height threshold of 0.25. Module-trait relationships were assessed by correlating module eigengenes with clinical traits (Normal, CD, UC). Hub genes were defined as genes with module membership (MM) > 0.8 and gene significance (GS) > 0.6.

### Functional enrichment analysis of DEGs and immune profile analysis

Functional enrichment analysis of DEGs was conducted using the “clusterProfiler” R package for Gene Ontology (GO) and Kyoto Encyclopedia of Genes and Genomes (KEGG) pathway analysis. Gene symbols were converted to Entrez IDs using the “org.Hs.eg.db” annotation package. KEGG analysis was performed using enrichKEGG() with organism=“hsa”, while GO analysis utilized enrichGO() with ont=“all” parameters.Statistical significance thresholds were defined as p < 0.05 for KEGG and GO analysis. Results were visualized using circular plots generated with the “circlize” and “GOplot” packages. Immune infiltration analysis utilized the CIBERSORT algorithm to quantify the relative fractions of 22 immune cell types across conditions (Normal, CD and UC) (10). Additionally, single-sample gene set enrichment analysis (ssGSEA) was conducted using the “GSVA” package for immune cell and hallmark pathway assessment. Spearman’s correlation analysis was employed to explore relationships between hub gene expression levels and immune characteristics.

### Machine learning for feature selection and model validation

Potential candidate genes were first identified by overlapping DEGs with genes from WGCNA hub modules, yielding seven hub genes for further analysis. Subsequently, two machine learning algorithms were applied for feature selection: least absolute shrinkage and selection operator (LASSO) implemented via the “glmnet” R package with 10-fold cross-validation, and random forest using the “randomForest” R package with 500 trees (11, 12). The intersection of genes selected by both LASSO and Random Forest algorithms resulted in a final four-gene signature. To evaluate the diagnostic performance of this four-gene signature, ten machine learning algorithms were implemented: Logistic Regression, Random Forest, Support Vector Machine (SVM), Naive Bayes, Linear Discriminant Analysis (LDA), Mixture Discriminant Analysis (MDA), Flexible Discriminant Analysis (FDA), Gradient Boosting Machine, XGBoost, and CatBoost. The dataset was randomly split into training (60%) and validation (40%) cohorts using createDataPartition() from the “caret” package. Model performance was assessed using sensitivity, specificity, accuracy, F1-score, and area under the ROC curve (AUC). To ensure robust evaluation, the analysis was repeated 10 times with different random training-validation splits, and performance metrics were averaged across iterations. ROC curves were generated using the “pROC” package for model comparison.

### Nomogram development based on the diagnostic biomarkers and treatment response prediction

Nomogram models were developed using the “rms” R package for dual purposes: predicting IBD diagnosis based on the key diagnostic genes, and predicting treatment responses to biologic therapies. For diagnostic prediction, logistic regression models were constructed with the four-gene signature as predictor variables and disease status as the response variable. Model effectiveness was evaluated through calibration curve analysis and decision curve analysis (DCA).

For treatment response prediction, separate nomogram models were developed using baseline gene expression data from four datasets: GSE92415 (golimumab), GSE16879 (infliximab), GSE206285 (ustekinumab), and GSE73661 (vedolizumab). Logistic regression models were fitted using the lrm() function with treatment response status as the dependent variable and four-gene expression levels as independent variables. Nomogram risk scores were calculated using the predict() function, and patients were stratified into high and low nomogram score groups based on median cutoff values. Treatment response rates were computed for each risk group and visualized using stacked bar plots generated with ggplot2. The predictive performance was evaluated across all biologic therapies to assess the generalizability of the four-gene signature for treatment response stratification.

### Consensus clustering and principal component analysis

Consensus clustering analysis was performed using the “ConsensusClusterPlus” R package to identify molecular subtypes based on the four hub gene expression patterns. The optimal cluster number was determined using the Proportion of Ambiguous Clustering (PAC) method. PCA was conducted using the “FactoMineR” package, and results were visualized using “ggplot2” with 95% confidence ellipses to demonstrate cluster separation.

### Acquisition and processing of scRNA-Seq data

The scRNA-Seq dataset for colon tissue was obtained from a previous investigation that included three discrete disease states: non-inflammatory, healthy, and inflammatory conditions (13). Specifically, the non-inflamed and inflamed colon samples were derived from patients diagnosed with CD, while the healthy samples were obtained from colon tissue of individuals without inflammation. Data preprocessing was conducted using the “Seurat” R package (v4.1.0). Low-quality cells and doublets were removed using quality control metrics, and the log2(CPM+1) transformation was applied to normalize the expression matrix for downstream analysis. Cells were initially categorized into three major compartments based on established marker genes: epithelial cells (CO_EPI; expressing EPCAM, KRT8, and KRT18), immune cells (CO_IMM; expressing CD45/PTPRC, CD3D, CD3G, CD3E, CD79A, CD79B, CD14, CD16, CD68, CD83, CSF1R, FCER1G), and stromal cells (CO_STR; expressing CDH5, COL1A1, COL1A2, COL6A2, and VWF). Each major compartment was subset and processed independently using the same pipeline: normalization with NormalizeData(), identification of highly variable features with FindVariableFeatures(), data scaling with ScaleData(), and principal component analysis with RunPCA(). Pre-computed UMAP coordinates from the original study were imported and applied to preserve the original dimensional reduction. Cell subtypes within each compartment were refined and renamed for consistency: epithelial cells were classified into Enterocytes, Enteroendocrine cells, Epithelial Cycling cells, Goblet cells, Paneth cells, Stem cells, and Tuft cells. Immune cells were classified into B cells, dendritic cells (DC), ILCs, Macrophages, Mast cells, Monocytes, NK cells, Plasma cells, CD4+ T cells, CD8+ T cells, Naive T cells, Tregs, and Immune Cycling cells. Stromal cells were classified into Endothelial cells, Fibroblasts, Glial cells, Lymphatics, Pericytes, and Stromal Cycling cells. Hub gene expression visualization was performed using FeaturePlot() and DotPlot() functions. For each gene, dot plot analysis calculated the percentage of expressing cells and average expression levels across cell types and disease conditions. Differential expression analysis within each compartment was performed using FindAllMarkers() with parameters: logfc.threshold = 0.25, min.pct = 0.1, and only.pos = TRUE. Functional enrichment analysis of cell-type-specific differentially expressed genes was conducted using the “clusterProfiler” R package. For each major cellular compartment (epithelial, immune, and stromal), gene symbols were converted to Entrez IDs using “org.Hs.eg.db”. Gene Set Enrichment Analysis (GSEA) was performed using gseGO() function with parameters: ont = ‘BP’, pvalueCutoff = 0.05, genes ranked by average log2 fold change. The top 100 significantly enriched GO Biological Process terms from each cell type were selected for pathway network visualization using the “pathExplore” R package with enrichmentNetwork() function.

### Identification of potential therapeutic drugs for treatment response enhancement

The Connectivity Map (CMap) database was utilized to identify compounds that could potentially improve treatment response in IBD patients (14). Treatment response molecular signatures were constructed using GSE16879 dataset by comparing infliximab responders versus non-responders, selecting the top 300 differentially expressed genes (150 upregulated, 150 downregulated). CMap scores were calculated using the eXtreme Sum (XSum) algorithm with topN=500. The top five compounds with the lowest CMap scores were identified as potential therapeutic agents for enhancing treatment efficacy.

### Prognostic analysis of key genes in CRC

The expression levels of key genes were analyzed between CRC and normal tissue samples to assess their prognostic value. Survival analysis was conducted using the “survminer” R package (v0.49), which determined the optimal cutoff value for each gene. Based on these cutoffs, samples were stratified into high- and low-expression groups, and survival curves were constructed.

### Evaluation of the therapeutic efficacy of MS.275 *in vivo*

Eight-week-old female C57BL/6 mice (n = 30) were procured from the Guangdong Medical Laboratory Animal Center and acclimatized to laboratory conditions for 7 days prior to the experiment. Acute colitis was induced by administering 2.5% dextran sodium sulfate (DSS) in drinking water for 7 days. Treatment groups were allocated as follows: TNF-α inhibitor group received intraperitoneal injections of infliximab (10 mg/kg) or isotype control IgG twice weekly, while the MS.275-treated group received daily intraperitoneal injections of MS.275 (20 mg/kg). On day 8, mice were sacrificed, and colonic tissues were harvested for histopathological analysis. The animal handling procedures were approved by the Animal Ethics Committee of Shenzhen Hospital, Southern Medical University (No.2022-0226).

### Statistical analysis

Statistical analyses were conducted using R software (version 4.1.0). The Wilcoxon rank-sum test was applied for non-normally distributed data, while independent samples t-tests were used for normally distributed variables. Comparisons among multiple groups were performed using the Kruska-Wallis test or one-way ANOVA. A two-tailed p-value < 0.05 was considered statistically significant.

## Results

### Differentially expressed genes identification in IBD and healthy control groups

**Figure 1:**
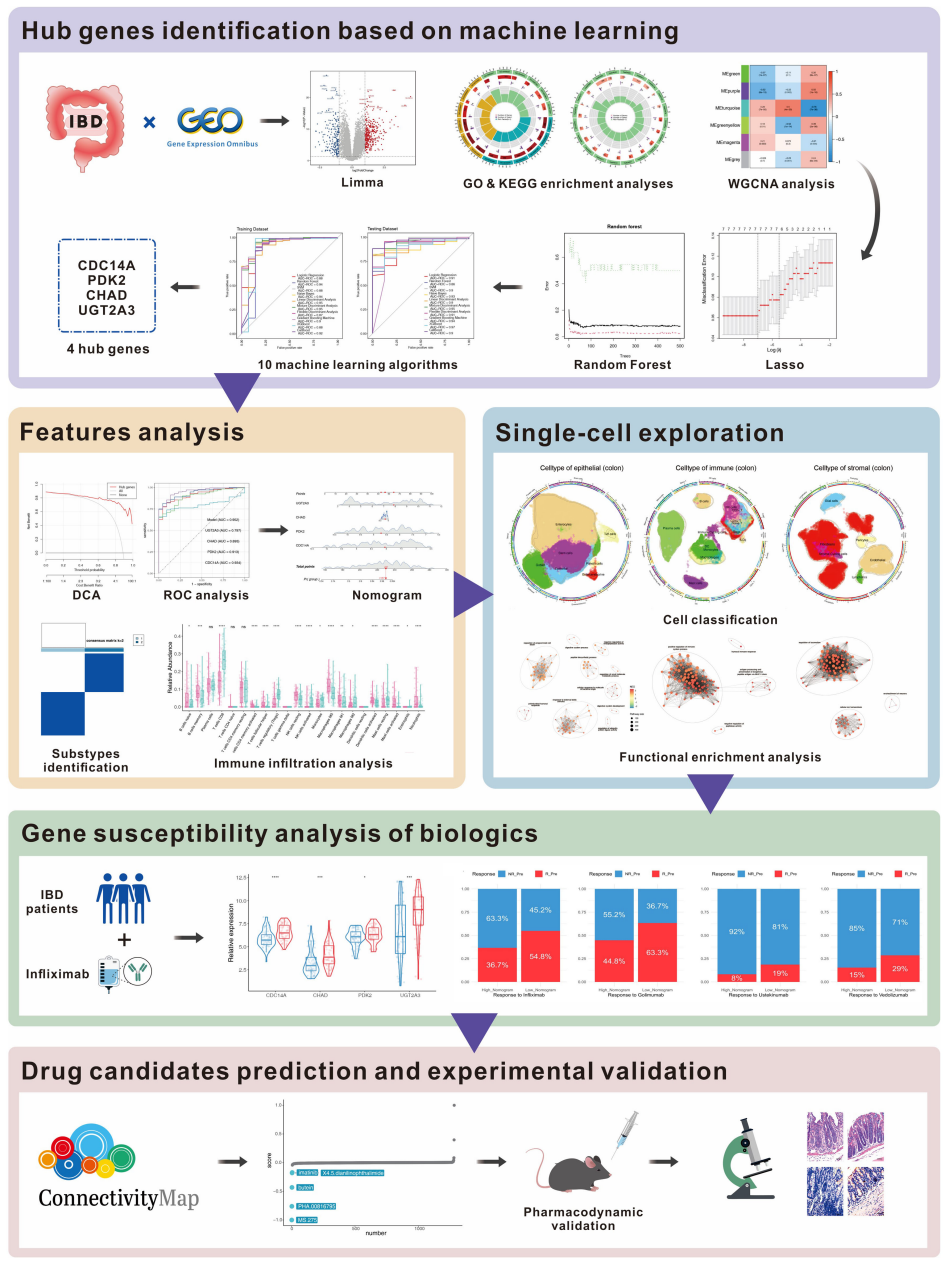
Workflow for multi-omics identification of IBD biomarkers and therapeutic targets. Multi-omics analysis workflow includes differential expression analysis (536 DEGs), WGCNA (6 co-expression modules), machine learning (4-gene signature), validation (AUC: 0.86-0.97), single-cell analysis (278,814 cells, 26 subtypes), treatment prediction, and drug discovery with experimental validation (MS.275). DEGs, differentially expressed genes; WGCNA, Weighted Gene Co-expression Network Analysis; AUC, Area Under the Curve.

**Figure 2:**
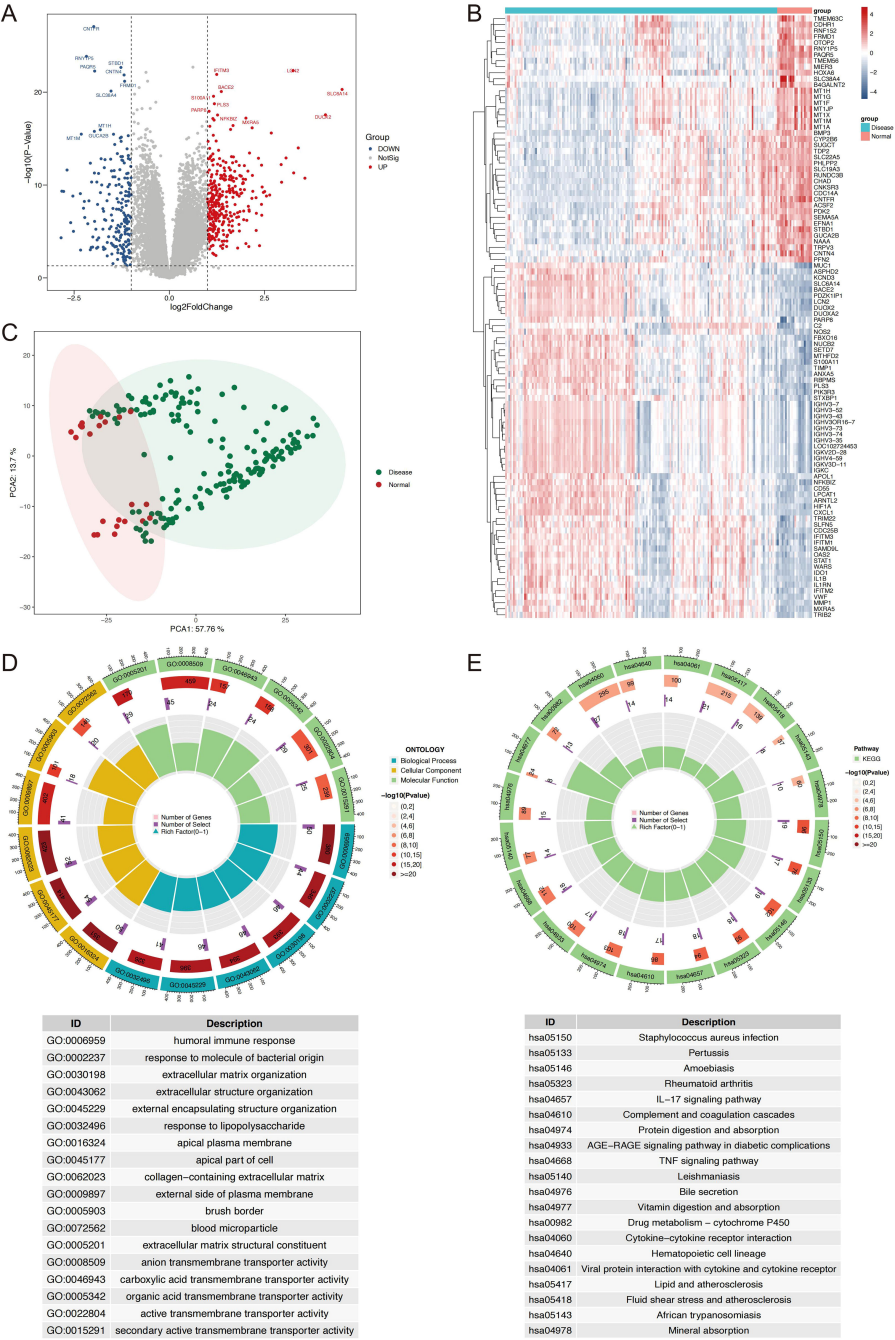
Identification and Characterization of DEGs in IBD vs. normal controls. **(A)** Volcano plot displaying DEGs, with upregulated (red) and downregulated (blue) genes based on log2 fold change (>1.0 or <-1.0) and P-value (<0.05). **(B)** Heatmap showing the expression profiles of the top 100 DEGs, with samples grouped by IBD and normal status. **(C)** PCA plot illustrating distinct clustering of IBD and HC samples based on gene expression data. **(D)** Circos plot of GO enrichment results, top 6 terms per ontology, biological process, cellular component, molecular function, are shown. **(E)** Circos plot of KEGG pathway enrichment results. DEGs, differentially expressed genes; IBD, inflammatory bowel disease; PCA, principal component analysis; HC, healthy controls; GO, Gene Ontology; KEGG, Kyoto Encyclopedia of Genes and Genomes.

The workflow of this study is outlined in **Figure 1**. A total of 536 differentially expressed genes (DEGs) were identified between IBD and HC samples, with 205 genes downregulated and 331 upregulated (**Figures 2A, B**). PCA demonstrated clear separation between IBD and HC groups based on gene expression profiles (**Figure 2C**). Functional enrichment analysis of DEGs using GO and KEGG revealed significant biological processes and pathways. GO analysis highlighted enrichment in processes such as humoral immune response, extracellular matrix organization, collagen-containing extracellular matrix, organic acid transmembrane transporter activity, and apical plasma membrane (**Figure 2D**). KEGG pathway analysis identified key pathways, including IL-17 signaling, AGE-RAGE signaling in diabetic complications, TNF signaling, drug metabolism-cytochrome P450, cytokine-cytokine receptor interaction, and viral protein-cytokine receptor interaction (**Figure 2E**).

### Co-expression network construction and hub module identification

**Figure 3:**
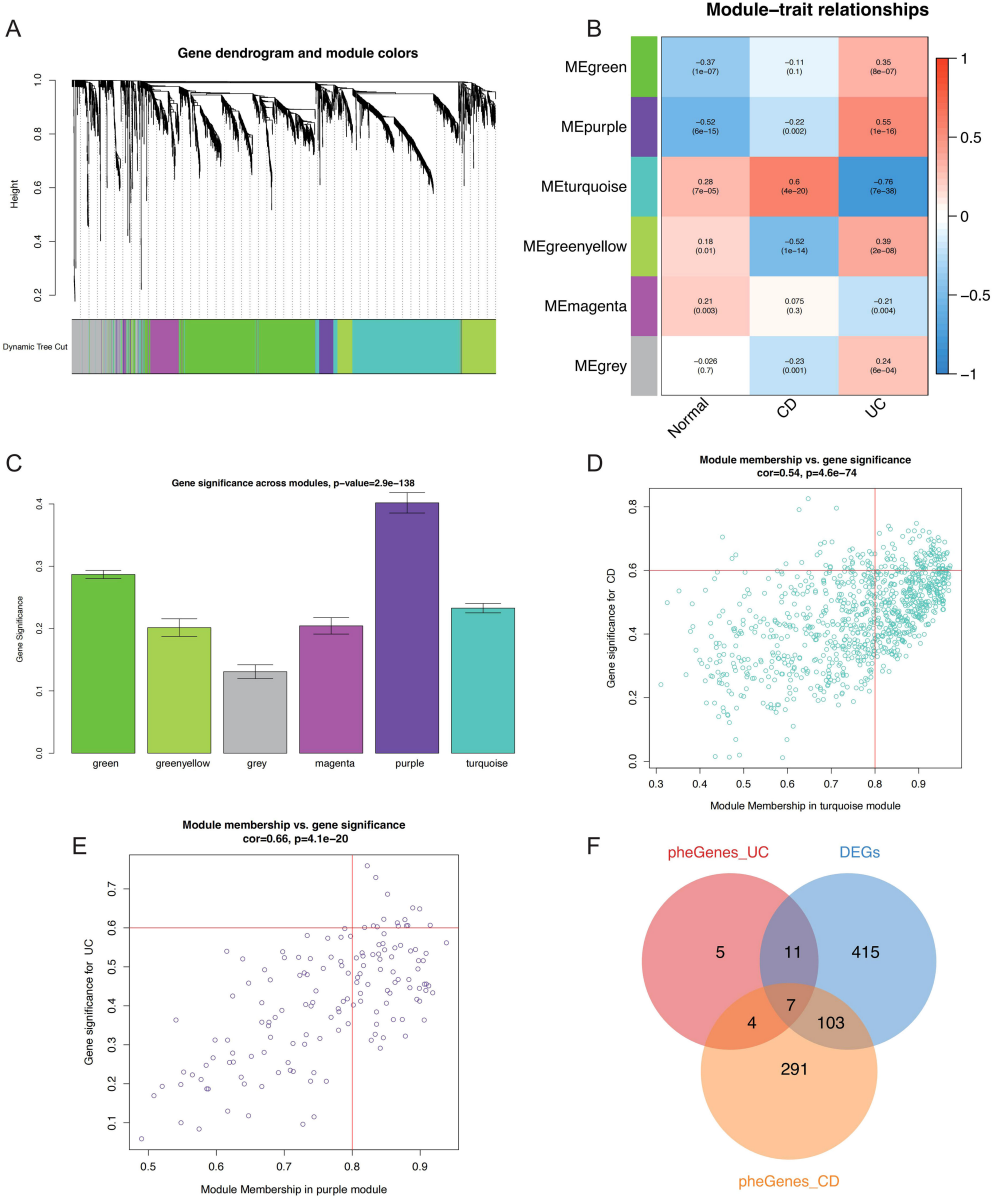
WGCNA-based identification of critical genes and modules associated with IBD. **(A)** Gene dendrogram and module color assignment showing the final six co-expression modules after dynamic tree cutting and module merging. **(B)** Heatmap displaying module-trait relationships between MEs and clinical phenotypes (Normal, CD, UC), with correlation coefficients and P-values annotated. **(C)** Bar plot showing module significance values (mean gene significance per module) across all six modules for IBD-related traits. Scatter plots illustrating the relationship between MM and GS for hub gene identification in the **(D)** turquoise module (CD-associated) and **(E)** purple module (UC-associated), with selection criteria of MM > 0.8 and GS > 0.6 indicated by red lines. **(F)** Venn diagram showing the intersection of DEGs, CD-related hub genes (turquoise module), and UC-related hub genes (purple module), identifying seven candidate hub genes for further analysis. WGCNA, Weighted Gene Co-expression Network Analysis; IBD, inflammatory bowel disease; MEs, module eigengenes; CD, Crohn’s disease; UC, ulcerative colitis; MM, module membership; GS, gene significance; DEGs, differentially expressed genes.

WGCNA was performed to explore co-expressed genes associated with IBD. To identify outliers and ensure data quality, sample clustering was performed using hierarchical clustering with average linkage method. A cutoff height of 12,000 was applied to remove outlying samples (**Supplementary Figure S1A**), and sample-trait relationships were visualized through heatmap analysis (**Supplementary Figure S1B**). For soft threshold selection, power values ranging from 1 to 20 were evaluated to achieve scale-free network topology. A soft threshold power of 11 was selected to balance scale-free topology model fit (R² > 0.8) and maintain reasonable mean connectivity, as determined by systematic evaluation of scale independence and mean connectivity plots (**Supplementary Figures S1C, D**). Gene co-expression networks were constructed using the topological overlap matrix (TOM) approach. Dynamic tree cutting was applied with a minimum module size of 80 genes and deepSplit parameter of 2 to identify initial gene modules (**Supplementary Figure S1E**). Similar modules were merged at a dissimilarity threshold of 0.25 to reduce redundancy (**Supplementary Figure S1F**), ultimately yielding six distinct co-expression modules (**Figure 3A**). Module-trait association analysis revealed significant correlations between specific modules and IBD phenotypes, with the turquoise module demonstrating strong positive correlation with CD (r = 0.6, P = 4×10^-20^) and the purple module showing significant positive association with UC (r = 0.55, P = 1×10^-16^) (**Figure 3B**). Module significance analysis across all six modules confirmed the biological relevance of turquoise and purple modules to IBD pathogenesis (**Figure 3C**). Hub genes within these disease-relevant modules were identified using stringent criteria of module membership (MM) > 0.8 and gene significance (GS) > 0.6, as illustrated in scatter plots for both turquoise and purple modules (**Figures 3D, E**). Venn diagram analysis integrating differentially expressed genes with hub genes from CD-associated (turquoise) and UC-associated (purple) modules identified seven candidate hub genes for subsequent machine learning-based feature selection (**Figure 3F**).

### Development and estimation of machine learning models

**Figure 4:**
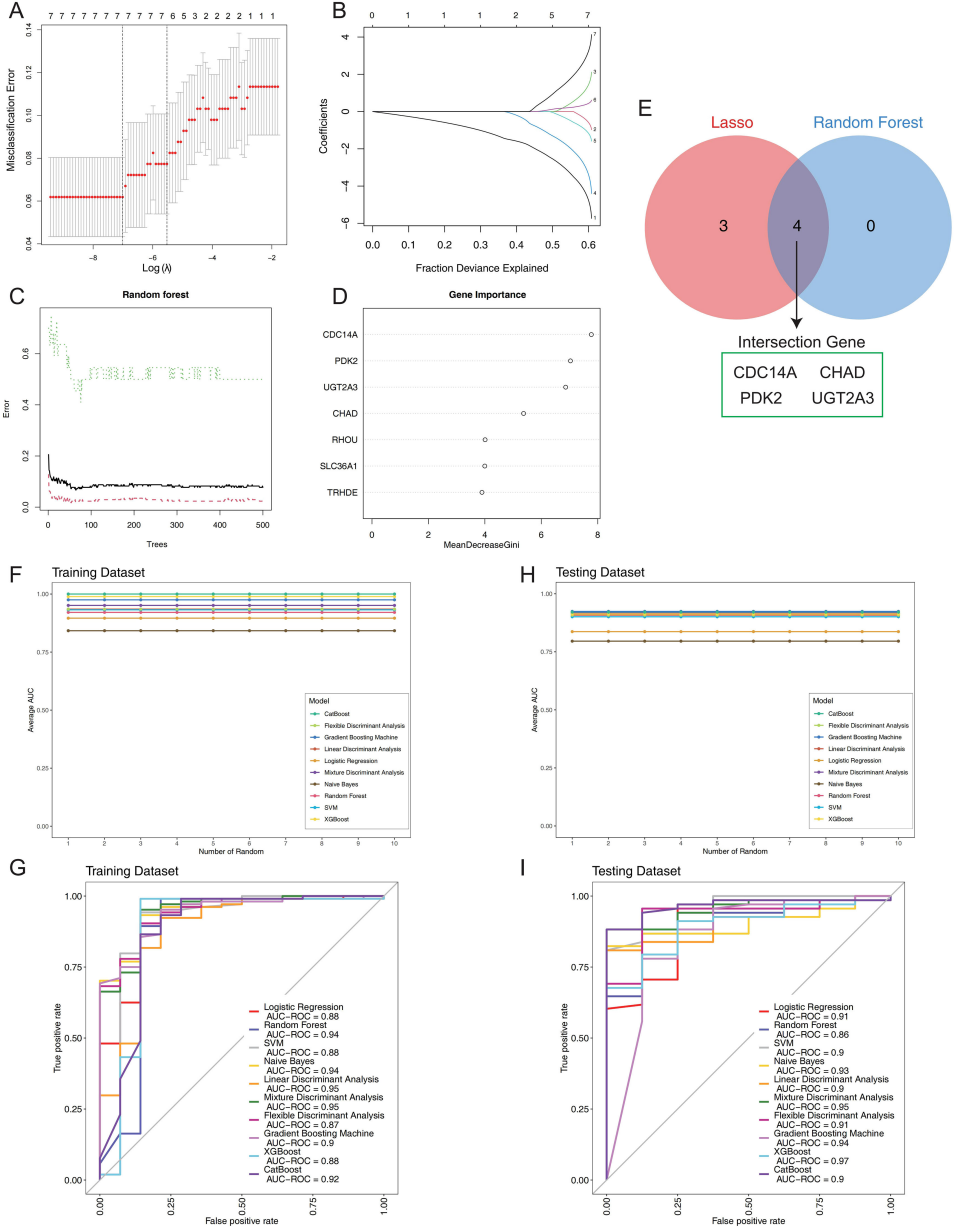
Construction and validation of the IBD prognostic model. **(A)** LASSO regression cross-validation optimization curve displaying misclassification error rates across different log(λ) values. **(B)** LASSO coefficient shrinkage path plot illustrating the progressive reduction of individual gene coefficients as the fraction of deviance explained increases. **(C)** Random forest error rate plotted against the number of classification trees. **(D)** Variable importance ranking derived from Random Forest analysis, showing mean decrease in Gini impurity for each gene. **(E)** Venn diagram comparing results from two machine learning algorithms. **(F)** Stability assessment of mean AUC values for 10 machine learning algorithms across 10 random training iterations on the training dataset (60% of GSE75214). **(G)** Comparative ROC curve analysis of 10 machine learning algorithms on the training dataset using 5-fold cross-validation. **(H)** Validation of model stability showing mean AUC values for all 10 algorithms across 10 random testing iterations on the independent validation set (40% of GSE75214). **(I)** ROC curves for the 10 classifiers on the test set, generated with 5-fold cross-validation. IBD, inflammatory bowel disease; LASSO, Least Absolute Shrinkage and Selection Operator; AUC, Area Under the Curve; ROC, Receiver Operating Characteristic.

To identify the most discriminative genes for IBD classification, we implemented a systematic two-stage feature selection strategy combining LASSO regression and Random Forest algorithms. LASSO regression with 10-fold cross-validation was applied first, where the optimal regularization parameter (λ) was systematically determined by minimizing misclassification error across the candidate gene pool (**Figure 4A**). The coefficient shrinkage path demonstrated the progressive selection process, with the cross-validation curve identifying the optimal lambda value that balanced model complexity and prediction accuracy (**Figure 4B**). This process selected seven genes with non-zero coefficients at the optimal regularization threshold. Complementarily, Random Forest analysis was performed using 500 classification trees to assess variable importance through Gini impurity reduction. The model achieved convergence with stable out-of-bag error rates, demonstrating robust performance (**Figure 4C**). Variable importance ranking revealed cell division cycle 14A (CDC14A) as the most discriminative feature, followed by pyruvate dehydrogenase kinase 2 (PDK2), UDP glucuronosyltransferase family 2 member A3 (UGT2A3), and chondroadherin (CHAD), with mean decrease in Gini scores clearly distinguishing these top-performing genes (**Figure 4D**). The systematic integration of both feature selection approaches through intersection analysis yielded a refined four-gene signature (CDC14A, PDK2, CHAD, UGT2A3), representing the consensus of both algorithms (**Figure 4E**).

To comprehensively evaluate the diagnostic performance of this four-gene panel, we implemented ten distinct machine learning algorithms: Logistic Regression, Random Forest, SVM, Naive Bayes, LDA, Mixture MDA, FDA, GBM, XGBoost, and CatBoost. Each algorithm was trained on 60% of the GSE75214 dataset and validated on the remaining 40%, with performance assessed across 10 random seed iterations to ensure statistical robustness and reproducibility. Performance stability analysis across multiple random iterations demonstrated exceptional model consistency (**Figures 4F, H**). In the training cohort, LDA and MDA achieved the highest AUCs of 0.95, followed by Random Forest and Naive Bayes (0.94), while FDA demonstrated the lowest performance (0.87) (**Figure 4G**). The testing cohort revealed superior generalization capabilities, with XGBoost achieving the highest AUC of 0.97, followed by MDA (0.95) and GBM (0.94). Remarkably, all ten algorithms demonstrated excellent discriminatory power with AUCs ranging from 0.86 to 0.97, confirming the robust diagnostic potential of the four-gene signature (**Figure 4I**). The comprehensive evaluation establishes that this systematically derived four-gene panel provides exceptional diagnostic accuracy for IBD classification, with XGBoost demonstrating optimal generalization performance and MDA showing consistent excellence across both training and validation cohorts.

### Establishment of nomogram based on characteristic genes to predict the risk of IBD

**Figure 5:**
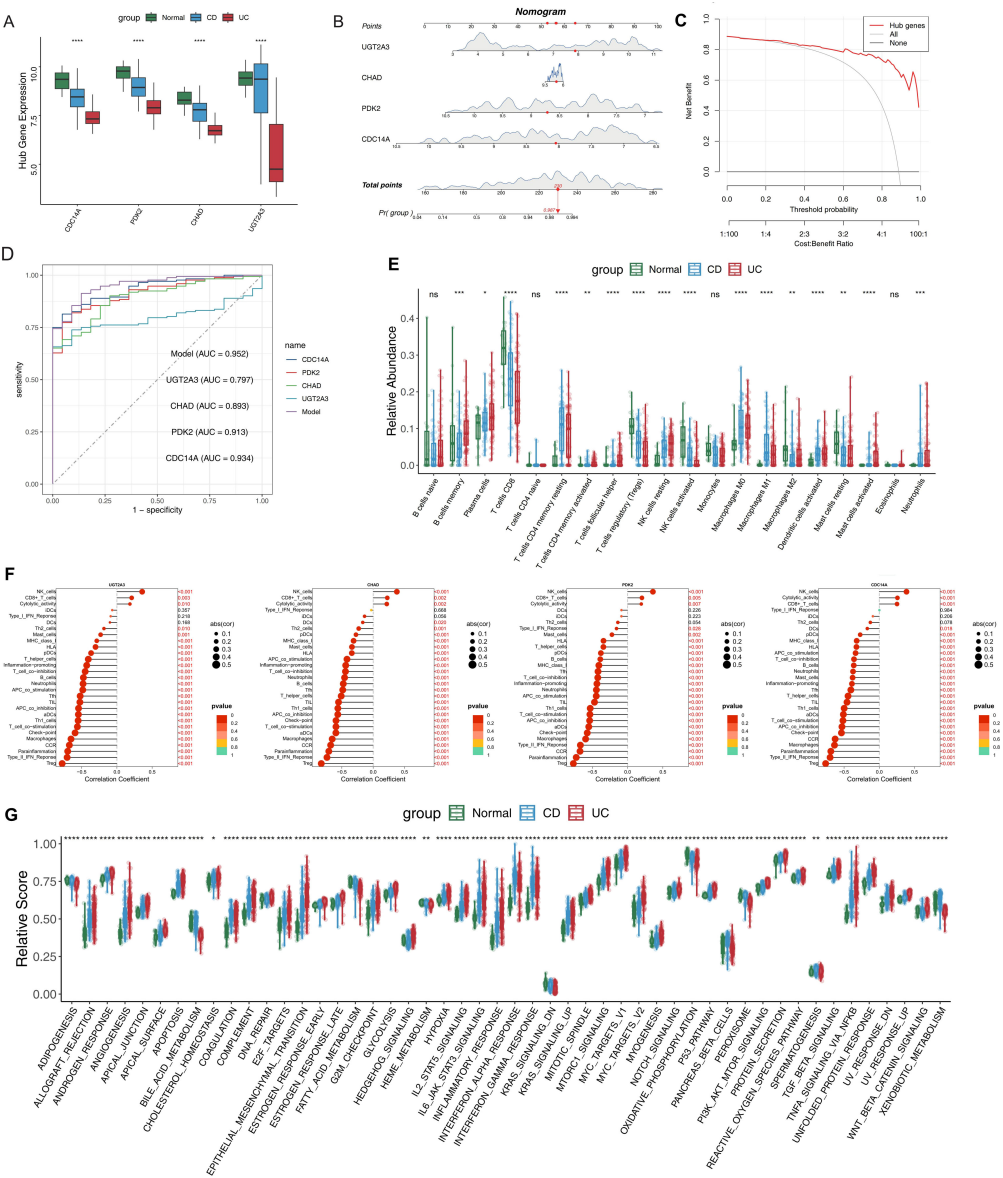
Clinical nomogram construction and comprehensive immune microenvironment characterization in IBD. **(A)** Expression levels of the four hub DEGs in IBD and normal samples. **(B)** Nomogram model constructed based on the four hub DEGs to predict the likelihood of IBD occurrence. **(C)** DCA curves evaluating the clinical utility of the nomogram model. **(D)** ROC curves for individual candidate genes and the nomogram model. **(E)** Relative proportions of immune cell subsets in IBD patients compared to normal controls. **(F)**Lollipop plots showing correlation coefficients between hub genes and immune features, including immune cell populations and functional pathways. Dot size represents correlation strength and color intensity indicates statistical significance. **(G)** Box plot highlighting differences in hallmark gene sets between IBD patients and normal controls. *p <0.05, **p <0.01, ***p<0.001, ****p<0.0001. IBD, inflammatory bowel disease; DEGs, differentially expressed genes; DCA, Decision Curve Analysis; ROC, Receiver Operating Characteristic. ns, not significant.

To validate the clinical relevance of our four-gene signature, we systematically analyzed the expression patterns of CDC14A, PDK2, CHAD, and UGT2A3 across UC, CD, and normal control samples. All four genes demonstrated statistically significant differential expression between IBD patients and healthy controls (**Figure 5A**). CDC14A, PDK2 and CHAD showed consistent downregulation in both CD and UC patients compared to normal controls, while UGT2A3 showed mild downregulation in CD, suggesting distinct regulatory mechanisms across disease subtypes. To develop a clinically applicable diagnostic tool, we constructed a nomogram incorporating these four genes. In this model, each gene was assigned a specific score, with cumulative points derived from all genes indicating varying levels of IBD risk (**Figure 5B**). DCA analysis confirmed that the nomogram provided significant clinical benefits to IBD patients, demonstrating its practical utility in clinical settings (**Figure 5C**). ROC analysis revealed excellent discriminatory performance for individual genes and superior performance for the integrated model (**Figure 5D**). Individual gene AUC values were: CDC14A (0.934), UGT2A3 (0.797), CHAD (0.893), and PDK2 (0.913). Remarkably, the integrated nomogram model achieved an AUC of 0.952, substantially exceeding any individual gene predictor and demonstrating exceptional diagnostic accuracy for IBD detection. To understand the immunological context of our gene signature, we conducted comprehensive immune profiling using CIBERSORT analysis, which revealed distinct patterns of immune cell composition between IBD patients and healthy controls (**Figure 5E**). Notable increases were observed in plasma cells, resting CD4+ memory T cells, resting NK cells, M0/M1 macrophages, and activated dendritic cells in both CD and UC patients. Conversely, CD8+ T cells, Tregs, activated NK cells, M2 macrophages, and resting mast cells showed decreased relative abundance in IBD patients compared to controls. Several immune populations including naïve B cells, naïve CD4+ T cells, monocytes, and eosinophils demonstrated no significant differences between groups. These findings confirm distinct inflammatory immune infiltration patterns characteristic of IBD pathogenesis. Correlation analysis revealed consistent patterns between all four hub genes and immune cell populations/functions (**Figure 5F**). Specifically, CDC14A, PDK2, CHAD, and UGT2A3 all showed significant positive correlations with NK cells, CD8+ T cells, and cytolytic activity. Conversely, all four genes demonstrated consistent negative correlations with the majority of other immune features, including Th1/Th2 cells, various DC subsets (DCs, pDCs, iDCs), macrophages, neutrophils, mast cells, B cells, T helper cells, and regulatory T cells. The genes also negatively correlated with immune functional pathways including inflammation-promoting activities, T cell co-stimulation/co-inhibition, APC co-stimulation/co-inhibition, checkpoint, MHC class I presentation, and CCR signaling. ssGSEA of hallmark pathways revealed extensive biological pathway alterations in IBD patients compared to normal controls (**Figure 5G**). Significantly upregulated pathways in IBD included inflammatory response, interferon-α and interferon-γ responses, IL6-JAK-STAT3 signaling, TNF-α signaling via NF-κB, and complement activation, reflecting the chronic inflammatory nature of IBD. We also observed significant activation of hypoxia response, glycolysis, and mTORC1 signaling pathways, indicating metabolic reprogramming in IBD tissues. Additionally, epithelial-mesenchymal transition, angiogenesis, and apoptosis pathways were significantly elevated, suggesting ongoing tissue remodeling and cellular stress responses. Notably, several pathways showed differential activation patterns between CD and UC patients, with UC generally displaying more pronounced pathway activation scores, particularly for inflammatory and metabolic pathways. These comprehensive pathway alterations provide mechanistic insights into IBD pathogenesis and highlight the potential regulatory roles of our four-gene signature in disease progression.

### Classification of colonic mucosal gene expression-driven subgroups and immune characteristics in IBD clusters

**Figure 6:**
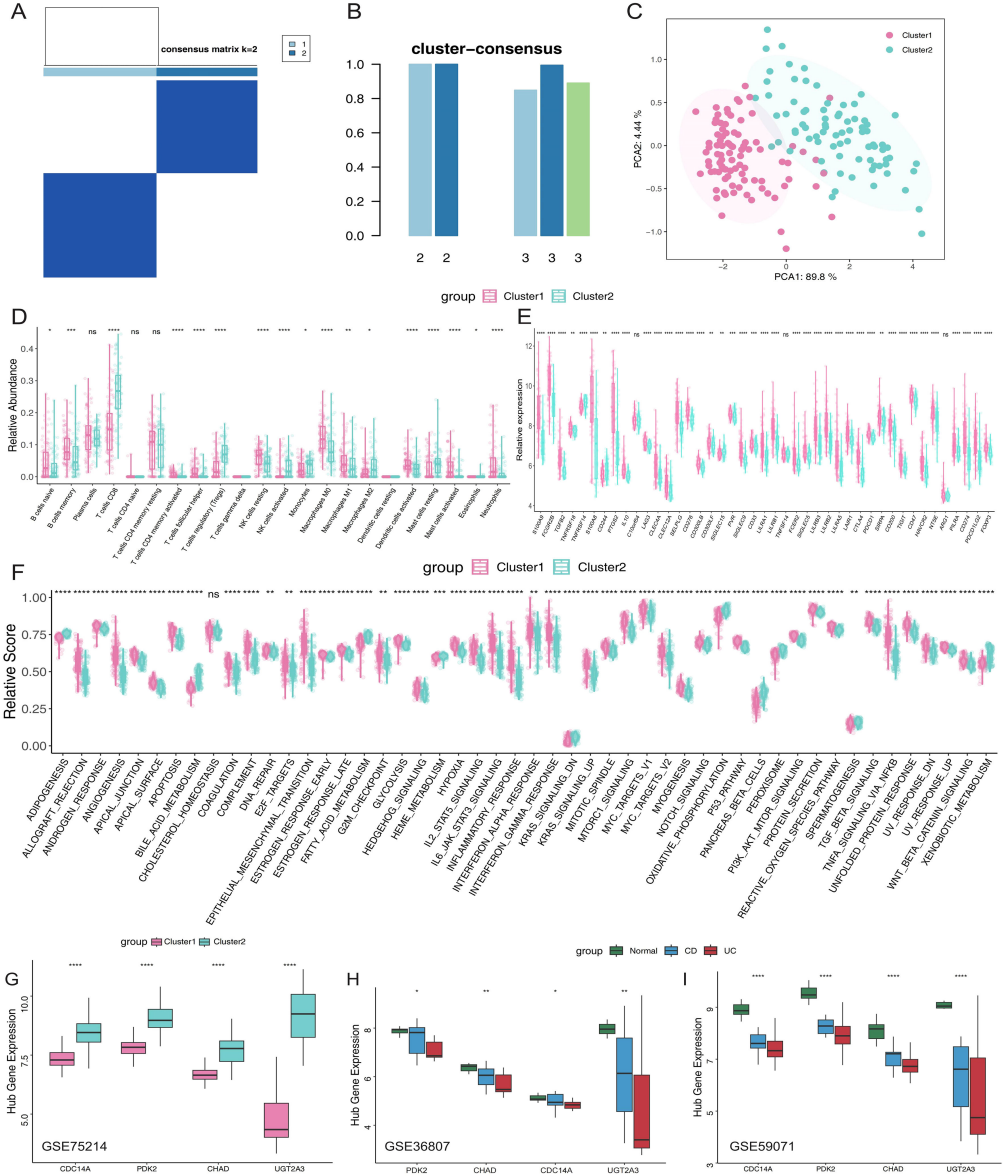
Molecular subtyping of IBD patients based on hub gene expression reveals distinct immune phenotypess. **(A)** Consensus clustering matrix when k =2. **(B)** Consensus clustering scores for k values of 2 and 3. **(C)** PCA analysis based on hub genes. **(D)** Proportions of immune cell subsets in Cluster 1 and Cluster 2. **(E)** Box plots illustrating differential expression of immune-related genes in Cluster 1 and Cluster 2. **(F)** Box plots comparing hallmark gene sets between the two clusters. **(G)** Relative expression levels of hub genes in different clusters. **(H, I)** External validation in independent datasets GSE36807 and GSE59071 confirming hub gene expression patterns across normal controls, CD, and UC patients. *p <0.05, **p <0.01, ***p<0.001, ****p<0.0001. IBD, inflammatory bowel disease; PCA, principal component analysis; CD, Crohn’s disease; UC, ulcerative colitis. ns, not significant.

To better understand the expression profile of hub genes in IBD, we employed a consensus clustering algorithm to classify 172 IBD samples based on the expression of four hub genes. We found when k=2, the consensus index of the CDF curve exhibited minimal fluctuation, and the consensus score was optimal, indicating that k = 2 provided a stable classification (**Figures 6A, B**). Furthermore, the findings of PCA revealed significant differences between the two clusters (**Figure 6C**). As a result, we split these IBD patients into two groups: cluster 1 (n=91) and cluster 2 (n=81). Subsequently, immune microenvironment characteristics were analyzed to assess the variations in infiltrating immune cells and immunological functionalities between the clusters. Cluster 1 exhibited higher levels of naive B cells, memory B cells, follicular helper T cells, resting NK cells, M0 macrophages, M1 macrophages, activated dendritic cells, activated mast cells, and neutrophils. Conversely, cluster 2 was enriched in CD8+ T cells, Tregs, activated NK cells, monocytes, M2 macrophages, and resting mast cells (**Figures 6D**), highlighting distinct immune profiles associated with each cluster. Analysis of immune-related gene expression revealed significant differences between the two IBD molecular subtypes (**Figure 6E**). The majority of analyzed genes showed statistically significant differential expression between clusters. Cluster 1 demonstrated significantly higher expression of multiple immune activation and inflammatory markers, including damage-associated molecular patterns (S100A9, S100A8), Fc gamma receptors (FCGR2B), and pro-inflammatory mediators (PTGS2). Additionally, Cluster 1 showed elevated expression of immune checkpoint molecules (LAG3, CTLA4, PDCD1, CD274, PDCD1LG2) and co-inhibitory receptors (TIGIT, HAVCR2), suggesting an immune-activated phenotype with concurrent regulatory mechanisms. Only a few genes (C100orf54, TNFSF14, ARG1) showed non-significant differences, confirming distinct immune microenvironments between the two molecular subtypes. To explore the underlying biological processes, GSVA analysis was conducted. In cluster 1, upregulated pathways included angiogenesis, epithelial-mesenchymal transition, hypoxia, glycolysis, inflammatory response, IL6-JAK-STAT3 signaling, mTORC1 signaling, TNFα signaling via NF-κB, and Wnt/β-catenin signaling. Conversely, adipogenesis, bile acid metabolism, oxidative phosphorylation, peroxisome function, and xenobiotic metabolism were downregulated (**Figure 6F**). Finally, differential expression of the four hub genes was assessed across the clusters. Cluster 2 showed significantly higher expression levels of these genes compared to cluster 1 (**Figure 6G**). These findings were validated using independent datasets (GSE36807 and GSE59071), confirming the observed expression patterns (**Figures 6H, I**).

### Expression profiles of hub genes at the single-cell level

**Figure 7:**
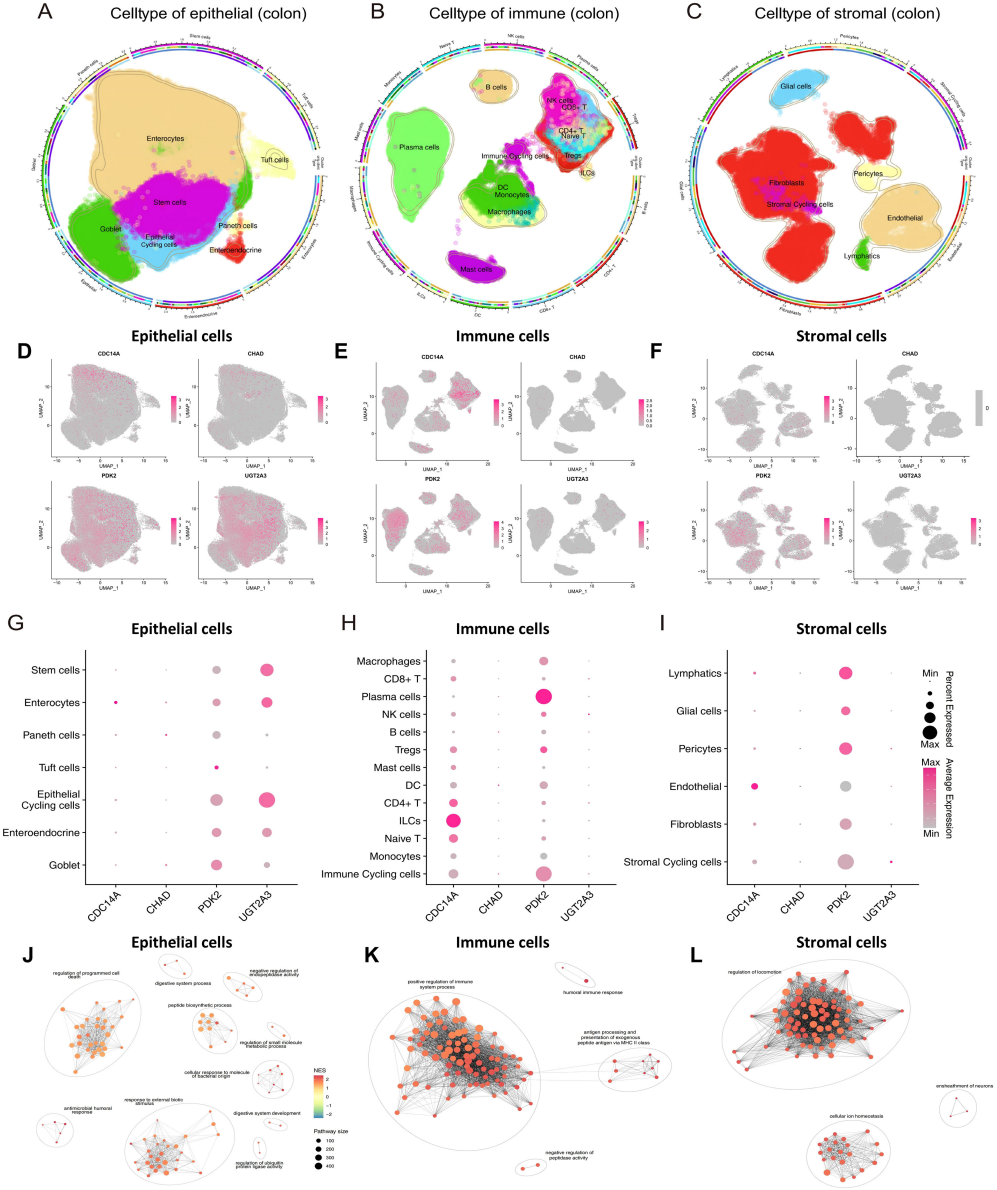
ScRNA-seq analysis reveals cell-type-specific expression patterns of hub genes in colonic tissues. **(A-C)** Circular UMAP plots showing the identification and distribution of cellular subtypes in epithelial **(A)**, immune **(B)**, and stromal **(C)** compartments from colonic tissues. Each cell type is color-coded and labeled, with the outer ring indicating disease conditions (healthy, non-inflammatory, inflammatory). **(D-F)** UMAP feature plots displaying the expression patterns of four hub genes across epithelial **(D)**, immune **(E)**, and stromal **(F)** cell compartments. Expression intensity is indicated by color gradient from gray (low/no expression) to pink/red (high expression). **(G-I)** Dot plots illustrating the expression levels and percentage of cells expressing each hub gene across different cell subtypes in epithelial **(G)**, immune **(H)**, and stromal **(I)** compartments. Dot size represents the percentage of expressing cells, and color intensity indicates average expression level. **(J-L)** Pathway enrichment networks for epithelial **(J)**, immune **(K)**, and stromal **(L)** cells generated using pathExplore package. Networks show the top 100 GO Biological Process terms from GSEA results (gseGO, pvalueCutoff < 0.05) for each cell type. Each node represents an enriched pathway, with node size reflecting pathway size (number of genes in pathway), and node color indicating normalized enrichment score (NES). Edges connect pathways with high gene set similarity, and pathway clusters are grouped with ellipses and labeled with representative biological themes. scRNA-seq, single-cell RNA sequencing; UMAP, Uniform Manifold Approximation and Projection; CDC14A, Cell Division Cycle 14A; CHAD, Chondroadherin; PDK2, Pyruvate Dehydrogenase Kinase 2; UGT2A3, UDP Glucuronosyltransferase Family 2 Member A3.

ScRNA-seq analysis encompassed 278,814 cells categorized into three major compartments: 97,345 epithelial cells, 142,045 immune cells, and 39,424 stromal cells, using cell-type markers as described in previous studies (13). Within the epithelial compartment, cells were classified into seven distinct subtypes: Enterocytes, Enteroendocrine cells, Epithelial Cycling cells, Goblet cells, Paneth cells, Stem cells, and Tuft cells. The immune compartment comprised thirteen subtypes: B cells, dendritic cells (DCs), innate lymphoid cells (ILCs), immune cycling cells, macrophages, mast cells, monocytes, NK cells, plasma cells, CD4+ T cells, CD8+ T cells, naive T cells, and regulatory T cells (Tregs). Stromal cells were subdivided into six subtypes: endothelial cells, fibroblasts, glial cells, lymphatics, pericytes, and stromal cycling cells (**Figures 7A-C**). UMAP visualization and dot plot analysis revealed distinct cell-type-specific expression patterns of our four hub genes across all cellular compartments (**Figures 7D-I**). PDK2 demonstrated the most widespread expression pattern, with particularly high levels in epithelial cycling cells, stem cells, goblet cells, and enterocytes within the epithelial compartment, suggesting a critical role in epithelial proliferation and metabolic regulation. Among immune cells, PDK2 was prominently expressed in plasma cells and immune cycling cells, while stromal cell types including pericytes, lymphatics, and stromal cycling cells also exhibited notable expression levels. UGT2A3 showed preferential expression in epithelial cells, with the highest levels observed in epithelial cycling cells, stem cells, and enterocytes, indicating its specialized involvement in xenobiotic metabolism and epithelial barrier function. This epithelial-restricted expression pattern aligns with the known role of UGT2A3 in intestinal detoxification processes. CDC14A displayed selective enrichment in innate lymphoid cells (ILCs) within the immune compartment, supporting its role in innate immune cell regulation and recruitment. This cell-type-specific expression pattern suggests CDC14A may serve as a critical regulator of ILCs-mediated immune responses in intestinal inflammation. CHAD exhibited modest but detectable expression across multiple cell types. However, the overall expression levels were lower compared to the other three hub genes. These single-cell expression patterns validate the biological relevance of our four-gene signature and provide mechanistic insights into their cell-type-specific functions in IBD pathogenesis, particularly highlighting their distinct roles in epithelial barrier maintenance (UGT2A3), metabolic regulation (PDK2), immune cell activation (CDC14A), and tissue remodeling processes (CHAD).

Functional enrichment analysis revealed distinct pathway associations for each cellular compartment (**Figures 7J-L**). GSEA was performed on each major cell type using gseGO, and the top 100 enriched GO Biological Process terms were visualized as pathway networks. In epithelial cells, the pathway network revealed interconnected clusters primarily associated with regulation of programmed cell death, digestive system process, negative regulation of endopeptidase activity, peptide biosynthetic process, regulation of small molecule metabolic process, cellular response to bacterial molecular patterns, antimicrobial humoral response, response to external biotic stimulus, regulation of ubiquitin protein ligase activity, and digestive system development. The network topology highlighted metabolic regulation and epithelial barrier function as central biological themes. In immune cells, the pathway enrichment network demonstrated distinct clusters enriched for positive regulation of immune system processes, humoral immune responses, antigen processing and presentation via MHC class II, and negative regulation of peptidase activity. The network structure emphasized immune activation and antigen presentation as predominant functional modules. In stromal cells, the pathway network revealed clusters predominantly associated with regulation of cellular locomotion, ensheathment of neurons, and cellular ion homeostasis. The network topology indicated tissue remodeling and neural support functions as key stromal cell activities. Each pathway network utilized similarity-based clustering to group related pathways, with cluster names automatically generated to represent the major biological themes within each cellular compartment.

### Hub gene expression profiles predict biologic treatment response and identify potential therapeutic targets

**Figure 8:**
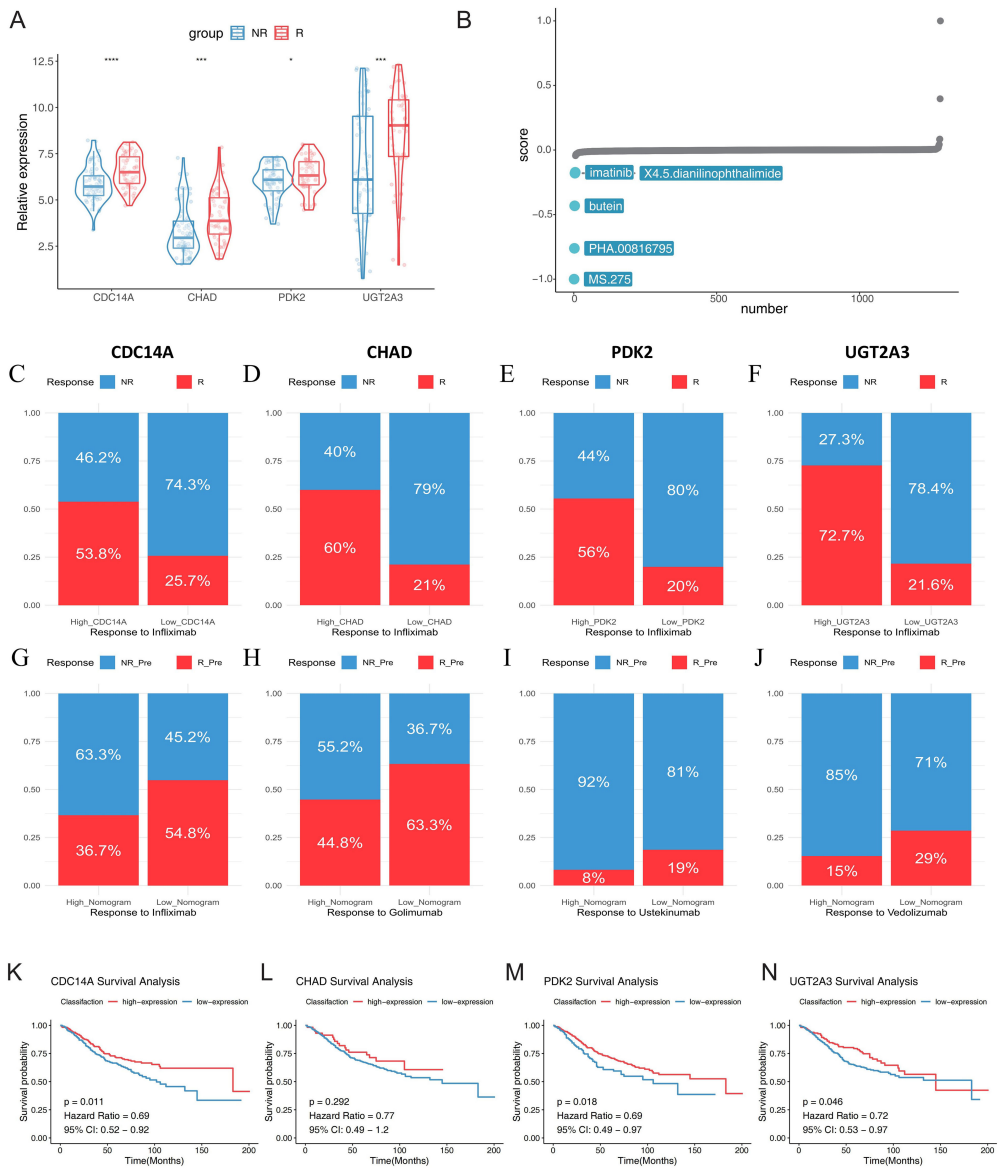
Hub gene expression predicts biologic treatment response and identifies potential therapeutic targets in IBD. **(A)** Violin plots showing expression levels of CDC14A, PDK2, CHAD, and UGT2A3 in infliximab non-responders (blue) versus responders (red) from GSE16879 dataset. **(B)** CMap analysis identifying potential therapeutic compounds for enhancing treatment response. **(C-F)** Stacked bar charts displaying response rates to infliximab treatment stratified by high versus low expression levels of each hub gene, R: mixed pre- and post-treatment responders in UC; NR: mixed pre-treatment and post-treatment non-responders in UC. **(G-J)** Treatment response rates for multiple biologic therapies based on nomogram scores derived from the four-gene signature using baseline samples: golimumab (GSE92415), infliximab (GSE16879), ustekinumab (GSE206285), and vedolizumab (GSE73661). Patients were stratified into high and low nomogram score groups, with response rates shown as percentages. **(K-N)** Kaplan-Meier survival curves for colorectal cancer patients (GSE39582) stratified by high and low expression levels of CDC14A, PDK2, CHAD, and UGT2A3, demonstrating prognostic significance. *p <0.05, ***p<0.001, ****p<0.0001. IBD, inflammatory bowel disease; NR, non-responders; R, responders; CMap, Connectivity Map;.

To investigate the clinical utility of our identified hub genes in predicting therapeutic responses, we analyzed their expression patterns across multiple biologic treatment datasets. Using the GSE16879 dataset, we first examined the relationship between the four hub genes (CDC14A, PDK2, CHAD, and UGT2A3) and infliximab treatment response in IBD patients. Violin plot analysis revealed significantly lower expression levels of all four hub genes in non-responders compared to responders to infliximab therapy (**Figure 8A**). To identify potential therapeutic compounds that could enhance treatment response in IBD patients, we employed CMap analysis using molecular signatures derived from treatment response patterns. By comparing gene expression profiles between infliximab responders and non-responders, we constructed a treatment response signature and screened for compounds capable of reversing the non-response molecular pattern. The analysis identified five promising small-molecule compounds with negative CMap scores, indicating potential to improve treatment efficacy: MS.275 (showing the strongest therapeutic potential), PHA.00816795, butein, imatinib, and X4.5.dianilinophthalimide (**Figure 8B**; **Supplementary Table 1**).

Further stratification analysis demonstrated that patients with high expression levels of each hub gene consistently showed markedly higher response rates to infliximab treatment. Especially in the UC samples of the GSE16879 dataset, high CDC14A, PDK2, CHAD, and UGT2A3 expression groups exhibited substantially greater proportions of treatment responders compared to their low expression counterparts (**Figures 8C-F**). These findings strongly suggest that elevated expression of these four genes enhances infliximab’s anti-inflammatory therapeutic efficacy. We also developed nomogram models incorporating all four hub genes to predict treatment responses across multiple biologics. Using logistic regression models, we calculated nomogram scores and stratified patients into high and low score groups based on median values. Remarkably, across all tested biologics, patients with low nomogram scores demonstrated consistently higher positive response rates. For TNF-α inhibitors, patients with low nomogram scores showed favorable response rates of 63.3% to golimumab and 54.8% to infliximab. For the integrin α4β7 inhibitor vedolizumab, patients with low nomogram scores achieved a 29% response rate, while the high nomogram score group showed only 15% response rate, indicating predictive value of the nomogram model. For the IL-12/23 inhibitor ustekinumab, although overall response rates were modest across both groups, the low nomogram score group (19%) still demonstrated superior responses compared to the high score group (8%) (**Figures 8G-J**). While sample size limitations precluded statistical significance testing, these findings highlight the potential of molecular fingerprints in guiding personalized biologic therapy selection. Finally, we investigated the prognostic significance of our hub genes in CRC using the GSE39582 dataset. Optimal expression cutoffs for survival analysis were determined using the surv_cutpoint function, and Kaplan-Meier survival curves were constructed to compare high and low expression groups. Survival analysis revealed that elevated expression levels of CDC14A (p = 0.011, HR = 0.69), PDK2 (p = 0.018, HR = 0.69), and UGT2A3 (p = 0.046, HR = 0.72) were significantly associated with improved overall survival in CRC patients. While CHAD showed a similar trend toward better prognosis with high expression (HR = 0.77), this association did not reach statistical significance (p = 0.292) (**Figures 8K-N**).

### MS.275 enhances the therapeutic efficacy of TNF-α inhibitor in DSS-induced colitis

**Figure 9:**
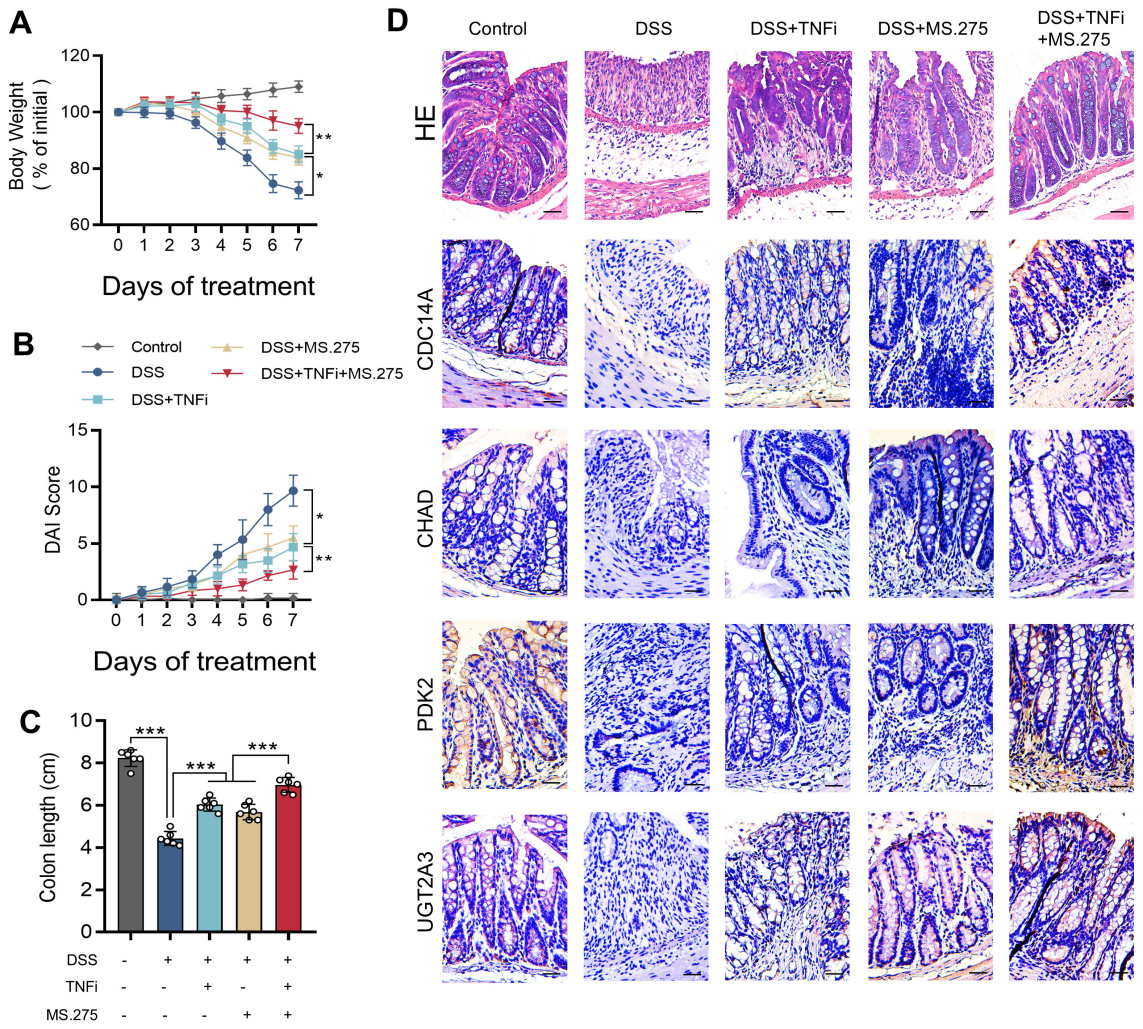
MS.275 enhances TNF-α inhibitor efficacy in DSS-induced colitis. **(A)** Body weight changes expressed as percentage of initial body weight over the 7-day experimental period (n=6 mice per group). **(B)** DAI scores during the experimental period. **(C)** Colon length measurements. **(D)** Representative H&E-stained colon sections, and immunohistochemical staining results of CDC14A, CHAD, PDK2 and UGT2A3. Scale bars = 50 μm. *p <0.05, ***p<0.001. TNF-α, tumor necrosis factor-alpha; DSS, dextran sodium sulfate; DAI, Disease Activity Index; H&E, hematoxylin and eosin; TNFi, TNF-α inhibitor.

To validate the therapeutic predictions from our CMap analysis and establish a mechanistic connection between our gene signature and treatment outcomes, we evaluated the efficacy of MS.275 in a DSS-induced colitis model using eight-week-old C57BL/6 mice exposed to 2.5% DSS for seven days, with concurrent administration of vehicle control, MS.275 alone, TNF-α inhibitor alone, or combination therapy. The rationale for combination therapy stems from MS.275’s ability to epigenetically reprogram inflammatory responses while TNF-α inhibitors directly block cytokine signaling, potentially creating synergistic anti-inflammatory effects through distinct but complementary molecular pathways. Disease severity assessment revealed that combination therapy provided superior therapeutic benefits, with mice receiving dual treatment showing significantly attenuated weight loss, lower DAI scores, and improved colon length preservation compared to monotherapy groups (**Figures 9A-C**). Histopathological analysis using H&E staining demonstrated that while DSS exposure resulted in severe mucosal damage with extensive ulceration and crypt destruction, combination therapy achieved near-complete restoration of normal colonic architecture with well-preserved crypts and minimal inflammatory infiltration (**Figure 9D**, top row). Mechanistically, the superior efficacy of combination therapy likely reflects MS.275’s ability to restore chromatin accessibility and promote transcription of protective genes while TNF-α inhibition reduces inflammatory cytokine-mediated tissue damage. Immunohistochemical analysis of our four hub genes preliminarily validated this epigenetic restoration, with each gene exhibiting cell-type-specific staining patterns that largely corresponded to their predicted functional roles. CDC14A immunostaining showed localization in scattered immune cells within the lamina propria and was markedly reduced following DSS exposure but robustly restored with combination therapy. UGT2A3 demonstrated predominant epithelial localization in surface and crypt epithelia with minimal stromal or immune staining, validating its selective expression in epithelial cells identified by single-cell analysis. PDK2 exhibited the most widespread tissue distribution across epithelial crypts, lamina propria immune infiltrates, and stromal elements, confirming its broad cellular expression profile and central metabolic regulatory role. DSS exposure dramatically suppressed expression of all four genes across their respective cellular compartments, while combination therapy achieved the most comprehensive restoration of gene expression patterns, establishing a direct correlation between therapeutic efficacy and molecular recovery. These findings demonstrate that MS.275 significantly enhances the efficacy of TNF-α inhibitors, possibly through the epigenetic restoration of protective gene expression programs. This supports the clinical potential of combining HDAC inhibitors with biologics for the treatment of refractory IBD. The mechanistic synergy between epigenetic modulation and cytokine inhibition provides a rational foundation for precision combination therapies in IBD management.

## Discussion

The integration of core mucosal molecular signatures into IBD management represents a transformative advancement in gastroenterology. Recent progress in multi-omics approaches has significantly enhanced our understanding of the molecular underpinnings of IBD pathogenesis, revealing intricate interactions between genetic susceptibility, immune dysregulation, and environmental triggers. This molecular characterization provides a robust foundation for precision medicine in IBD care, where treatment decisions can be guided by individual patient profiles rather than conventional symptom-based classifications. Our four-gene signature (CDC14A, PDK2, CHAD, and UGT2A3) exemplifies this approach by capturing key aspects of immune homeostasis and epithelial barrier function that are central to IBD pathophysiology. This evolution in IBD management represents a paradigm shift from conventional symptom-based approaches toward molecular-guided therapeutic strategies, aiming to harness patient-specific characteristics to predict disease trajectory and optimize treatment selection (15). Despite substantial progress, significant knowledge gaps persist regarding reliable biomarkers for longitudinal prediction of disease progression and treatment response. The clinical interpretation of molecular signatures requires rigorous validation across multiple dimensions—diagnostic accuracy, prognostic value, and therapeutic predictive capacity, all of which we have addressed through our comprehensive analytical framework.

Our transcriptomic analysis identified 536 DEGs (205 downregulated, 331 upregulated) in IBD tissues compared to healthy controls, revealing significant dysregulation in pathways critical to IBD pathophysiology. Functional enrichment analysis highlighted key biological processes including humoral immune response, extracellular matrix organization, collagen-containing extracellular matrix, organic acid transmembrane transporter activity, and apical plasma membrane components. KEGG pathway analysis identified critical signaling cascades with established roles in IBD pathogenesis, including IL-17 signaling, AGE-RAGE signaling in diabetic complications, TNF signaling, drug metabolism-cytochrome P450, cytokine-cytokine receptor interaction, and viral protein-cytokine receptor interaction. The IL-17 pathway has been established as a critical mediator in CD pathogenesis, where elevated IL-17 expression stimulates intestinal fibroblasts to increase production of NFKBIZ and CXCL1 (16). Similarly, our identification of AGE-RAGE signaling pathway dysregulation corroborates findings that RAGE activation induces intestinal inflammation through enhanced oxidative stress and endothelial activation, effects that can be mitigated by RAGE-specific inhibitors (17). The dysregulation of cytochrome P450 metabolism further supports evidence that specific CYP enzymes are required for gut barrier function enhancement and colitis protection (18). These pathway-level findings provide important molecular context for our four-gene signature and reinforce the biological relevance of the identified biomarkers in IBD pathogenesis and therapeutic response prediction.

WGCNA analysis revealed six distinct co-expression modules, with the turquoise module demonstrating strong correlation with CD and the purple module with UC. Through systematic intersection of DEGs with disease-specific modules, we identified seven hub genes for further machine learning refinement. The strategic integration of LASSO regression and Random Forest algorithms yielded our final four-gene signature, which demonstrated exceptional diagnostic performance across ten distinct machine learning algorithms with AUC values ranging from 0.86 to 0.97. XGBoost achieved optimal performance (AUC=0.97), while the integrated nomogram model incorporating all four genes achieved superior accuracy (AUC=0.952) compared to individual gene predictors: CDC14A (AUC=0.934), PDK2 (AUC=0.913), CHAD (AUC=0.893), and UGT2A3 (AUC=0.797). The identified hub genes demonstrate distinct associations with IBD pathophysiology. CDC14A, a dual-specificity phosphatase family member, primarily functions in reversing CDK-dependent phosphorylation events and regulates CDC25 activity at the G2/M transition (19). Chen et al. (20) revealed novel CDC14A functions in cell migration and adhesion, distinct from its mitotic roles. Given that intestinal mucosal barrier dysfunction represents a fundamental pathogenic mechanism in IBD, CDC14A-mediated epithelial cell regulation may offer therapeutic potential for enhancing barrier function. PDK2, the most ubiquitously expressed PDK isoform, exhibits regulation by nuclear hormone receptors and plays crucial roles in metabolic reprogramming and inflammatory responses (21, 22). Recent investigations have demonstrated that PDK inhibition suppresses Th2 cell development (23), while PDK activation promotes macrophage M1 polarization and NLRP3 inflammasome activation (24, 25). Furthermore, PDK2 influences LPS-induced endotoxin shock by modulating TLR4-mitogen-activated protein kinase signaling and augmenting proinflammatory cytokine production in macrophages and DCs (26). PDK4 deletion in CD4+ T cells attenuates colitis through metabolic regulation (27), underscoring the need for comprehensive investigation of PDK2’s immunometabolic role in IBD. The roles of CHAD and UGT2A3 in IBD pathogenesis remain less characterized, with CHAD functions primarily established in bone metabolism and cell-cell adhesion (28) (29), while UGT2A3 research has focused on cancer and drug metabolism (30, 31), leaving their IBD-specific contributions largely unexplored.

To comprehensively characterize the IBD immune microenvironment, we employed CIBERSORT and ssGSEA computational algorithms to quantify immune cell composition and immunological pathway activity from bulk transcriptomic data. These computational deconvolution methods provide a cost-effective and scalable approach for systematic immune profiling across large patient cohorts, offering valuable insights into disease-associated immune microenvironment dynamics as an alternative to experimental approaches such as flow cytometry or scRNA-seq. Our analysis revealed significant alterations in immune cell populations within IBD tissues, with markedly increased infiltration of M1 macrophages, neutrophils, activated DCs, and plasma cells compared to healthy controls. These findings align with previous research documenting extensive plasma cells infiltration in both CD and UC (32). Notably, it has been identified that IBD-related inflammation features mucosal accumulation of cytotoxic, granzyme B-expressing CD19+ and IgA+ cells, suggesting their involvement in IBD-associated epithelial damage (33). Proteomic and microscopic analyses have confirmed elevated levels of neutrophil and neutrophil extracellular trap (NETs)-associated proteins in UC samples, findings corroborated by multiple studies (34, 35). Wang et al. further demonstrated that NETs production increases significantly in DSS-induced colitis models, correlating with disease severity markers. Importantly, Cl-amidine treatment or PAD4 genetic deletion attenuated clinical colitis indices, intestinal inflammation, and barrier dysfunction (36). Pediatric IBD patients exhibit increased populations of activated mucosal macrophages with pronounced pro-inflammatory characteristics, marked by elevated expression of TNF-α, IL-1β, IL-6, and iNOS (37). The DCs alterations we observed reflect the complex pathophysiology of IBD, where these antigen-presenting cells congregate in specific gut locations including Peyer’s patches, isolated lymphoid follicles, and gut-associated lymphoid tissues. In IBD, colonic DCs exhibit abnormal immature phenotypes with altered homing marker expression. Intestinal DCs isolated from patients with UC demonstrate elevated levels of CCR9 and β7 integrin, but reduced levels of cutaneous lymphocyte antigen (CLA) and CCR4. When comparing DCs from healthy individuals to those from patients with CD, it is observed that the mucosa of CD exhibits elevated expression of CD40 and increased release of IL-6 and IL-12 (38, 39). While computational deconvolution provides valuable insights into immune microenvironment composition, several inherent limitations must be acknowledged. The accuracy of cell proportion estimates depends heavily on reference expression matrices that typically derive from peripheral blood or purified cells under resting conditions, potentially failing to capture the full heterogeneity or activation states of immune populations in inflamed tissues. Furthermore, bulk RNA-seq data represent averaged signals from mixed-cell populations, which may obscure contributions from rare cell subsets or transcriptionally similar populations, particularly relevant in IBD where dynamic immune infiltration and cellular plasticity complicate deconvolution accuracy. The resolution of closely related cell types, such as T helper cell subtypes with overlapping gene expression programs, remains inherently limited. Despite these limitations, our computational approach successfully identified immune signatures consistent with established IBD pathophysiology and provided a foundation for understanding how our four-gene signature correlates with specific immune cell populations and functional pathways.

The relationship between immune responses and cellular metabolism warrants particular attention in IBD pathophysiology. Our findings demonstrate that IBD patients exhibit enhanced glycolysis, hypoxia, ROS, and mTORC1 pathway activation, concurrent with suppressed oxidative phosphorylation (OXPHOS) signaling. This metabolic signature aligns with established patterns wherein proinflammatory responses correlate with increased mTORC1 and HIF-1α activity (40). Specifically, mTORC1 upregulation enhances HIF-1α expression, subsequently modulating glycolytic enzymes including lactate dehydrogenase A, PKM2, and hexokinase 2. Our findings underscore the significance of innate immune cell infiltration in IBD pathogenesis, characterized by elevated immune checkpoint activation, HLA gene expression, and immune response activation. The identified hub genes (UGT2A3, PDK2, CHAD, and CDC14A) demonstrated robust correlations with immune cell infiltration, suggesting their potential role in both the pathogenesis and diagnosis of IBD through inflammatory-immune pathway interactions.

In our study, we conducted consensus clustering using the ConsensusClusterPlus package with 1000 iterations of resampling (80% item subsampling) to ensure robustness. To assess cluster stability across different values of k, we examined multiple stability metrics including the consensus matrix heatmaps, cluster-consensus scores, and the PAC values. Specifically, the PAC metric was calculated for each k to quantify the dispersion of consensus values within the intermediate interval. The lowest PAC value was observed at k = 2, indicating optimal cluster stability and separation. We also computed the item-consensus scores and confirmed that clusters were highly consistent across resampling iterations. To validate the robustness of clustering, we additionally visualized sample distribution using PCA, which showed clear separation between subtypes. These analyses collectively identified k = 2 as the optimal clustering solution, revealing two distinct IBD molecular subtypes with unique immunological signatures. Compared to subtype-2, the modified subtype 1 exhibits enhanced immune activation as well as increased expression levels of HLA genes and immune checkpoints. Subtype-1, for instance, is more activated in the well-known signaling pathway comprising PI3K-AKT-mTOR, IL2-STAT5, and IL6-JAK-STAT3. The mammalian target of rapamycin (mTOR), a central regulator of nutrient sensing and cellular growth, plays a crucial role in intestinal macrophage polarization (40). Notably, the FDA-approved drug rapamycin, which directly targets mTOR, has demonstrated efficacy in reducing IBD-associated inflammation and improving inflammatory phenotypes (41). While mTOR represents a promising therapeutic target in IBD, comprehensive clinical studies are needed to validate the effectiveness of mTOR inhibitors. This molecular subtype classification provides valuable insights into immune regulatory mechanisms, potentially enabling more precise therapeutic approaches based on molecular rather than purely clinical phenotypes. The distinct inflammation-related pathway signatures between subtypes warrant further experimental investigation to elucidate their relationship with hub DEGs and their role in IBD pathogenesis.

ScRNA-seq analysis uncovered distinct cell-type-specific expression patterns that provide critical insights into gene function. Our results demonstrate that PDK2 exhibits broad expression distribution across multiple cell types, with particularly elevated expression levels observed in epithelial cycling cells, stem cells, goblet cells, and enterocytes. Additionally, significant PDK2 expression was detected in plasma cells and immune cycling cell populations. This expression profile strongly suggests PDK2 plays pivotal roles in both epithelial proliferation processes and immune system regulation. These findings are in complete agreement with published data, providing robust validation that PDK2 shows widespread expression throughout epithelial, immune, and stromal cell compartments. The concordance between our scRNA-seq results and existing literature substantially strengthens the evidence for PDK2’s fundamental involvement in these biological systems. UGT2A3 demonstrated preferential epithelial localization, particularly in cycling cells, stem cells, and enterocytes, reflecting its specialized role in intestinal detoxification processes. This expression pattern corresponds well with established tissue distribution data showing UGT2A3 as selectively enriched in the intestinal tract, with small intestine expression exceeding liver levels (31). The epithelial-specific expression of UGT2A3 underscores its potential as a biomarker for epithelial barrier integrity and metabolic dysfunction in inflammatory conditions. The cellular specificity observed for CDC14A offers further mechanistic insights into its role in the pathogenesis of IBD, particularly in mucosal immune regulation mediated by innate lymphoid cells. Notably, our observation of CHAD expression in epithelial and immune cells contradicts established expression patterns, as CHAD is known to be a cartilage matrix protein with highly restricted expression in chondrocytes, bone tissue, and connective tissue fibroblasts. Importantly, the integration of single-cell expression data with bulk tissue analysis strengthens the biological foundation of our gene signature. The cellular heterogeneity revealed by scRNA-seq analysis explains the diagnostic power of our four-gene panel, as each gene captures distinct aspects of IBD pathophysiology: metabolic dysregulation (PDK2), epithelial barrier dysfunction (UGT2A3), cell cycle perturbation (CDC14A), and extracellular matrix remodeling (CHAD). This multi-dimensional approach to biomarker development represents an advancement over single-pathway focused signatures and provides a more comprehensive molecular portrait of IBD complexity. Future studies should focus on validating these cellular expression patterns through spatial transcriptomics and immunohistochemistry to better understand the spatial organization of our signature genes within the inflamed intestinal architecture. Such approaches will facilitate the development of precision medicine strategies that account for both cellular heterogeneity and tissue-specific expression dynamics in IBD management.

Our nomogram-based risk stratification effectively predicted biologic therapy responses across multiple drug classes. Low nomogram score patients demonstrated superior response rates: 63.3% to golimumab, 54.8% to infliximab, and remarkably, 29% to vedolizumab compared to proportions in high-score patients. Even for ustekinumab, low-score patients showed better responses (19% vs. 8%). CMap analysis identified MS.275, a selective histone deacetylase (HDAC) inhibitor known to suppress inflammation and modulate T cell, B cell, and myeloid cell activities across multiple disease settings, as the top therapeutic enhancer for IBD treatment (42). Experimental validation confirmed synergistic effects between MS.275 and TNF-α inhibitors in DSS-induced colitis, significantly improving disease activity indices, colon length preservation, and restoring signature gene expression patterns through immunohistochemical analysis. The anti-inflammatory properties of this HDAC inhibitor, combined with its ability to modulate immune cell function, provide a mechanistic rationale for the observed therapeutic synergy and support its potential as an adjunctive therapy in IBD management. The clinical feasibility and translational potential of MS.275 in IBD management is highly promising given its established safety profile in clinical trials for hematological malignancies and solid tumors, with manageable adverse effects and oral bioavailability facilitating outpatient administration suitable for chronic IBD management. The synergistic combination targets distinct but complementary pathways, epigenetic regulation and inflammatory cytokine blockade, potentially allowing dose reduction while maintaining efficacy and minimizing toxicity risks. Future research priorities should focus on comprehensive mechanistic characterization of CHAD and UGT2A3, whose IBD roles remain incompletely understood despite robust diagnostic performance, through targeted functional studies investigating CHAD’s roles in epithelial cell adhesion and barrier integrity, and UGT2A3’s functions in xenobiotic metabolism and epithelial homeostasis. Patient stratification strategies should be refined through prospective validation studies incorporating real-time gene expression monitoring, longitudinal immune profiling, and machine learning-enhanced prediction algorithms to develop dynamic scoring systems adaptable to disease evolution and treatment responses.

The clinical implications of IBD extend beyond inflammatory manifestations to include increased risk of malignancy. Chronic inflammation associated with IBD elevates the risk of intestinal lymphoma, small bowel adenocarcinoma, CRC, and anal cancer, significantly impacting patient survival and quality of life. According to population-based studies, patients with UC who are older than 35 years of age have a 30% increased risk of CRC (43, 44). Consequently, we conducted additional investigations into the involvement of the identified hub genes in IBD and their potential impact on the prognosis of CRC. Our investigation of CRC prognosis revealed that elevated expression of CDC14A, PDK2, and UGT2A3 was significantly associated with improved overall survival, while CHAD showed similar trends without reaching statistical significance. These findings highlight the potential dual utility of our signature in both IBD management and cancer risk assessment. However, these findings require further validation through comprehensive studies to fully elucidate their prognostic significance.

While this study provides valuable insights into IBD pathogenesis and therapeutic targets, several limitations merit consideration. The model development relied primarily on publicly available datasets, necessitating comprehensive prospective clinical validation in diverse patient populations. Limited clinical metadata availability constrained our ability to account for disease duration, medication history, and demographic factors. Detailed mechanistic investigation of identified genes through *in vitro* and *in vivo* studies remains essential for enhancing predictive accuracy and elucidating their contributions to IBD pathogenesis.

## Conclusion

This study establishes a four-gene signature (CDC14A, PDK2, CHAD, UGT2A3) that effectively stratifies IBD patients and predicts treatment responses across multiple biologic therapies. Machine learning validation across ten algorithms demonstrated robust diagnostic performance, while experimental validation showed MS.275 enhances TNF-α inhibitor efficacy with concurrent restoration of signature gene expression. While these findings establish strong associations between the gene signature and IBD outcomes, mechanistic roles require further validation through targeted functional studies and preclinical modeling. For clinical translation, this signature could be implemented through routine mucosal biopsy analysis using standardized real-time reverse transcriptase-polymerase chain reaction platforms integrated with existing diagnostic workflows. Future studies should focus on mechanistic validation of individual hub genes and prospective clinical validation to establish evidence-based precision medicine applications in IBD management.

## Data Availability

The original contributions presented in the study are included in the article/**Supplementary Material**. Further inquiries can be directed to the corresponding authors.

## Supplementary material

Supplementary Figure 1 WGCNA gene co-expression network construction and module identification. **(A)** Sample clustering dendrogram based on Euclidean distance and average linkage method, with a cutoff height of 12,000 applied to remove outlier samples. **(B)** Sample dendrogram and trait heatmap showing the relationship between sample clustering and clinical traits (Normal, CD, UC), with binary trait encoding (1 = present, 0 = absent). **(C)** Scale independence plot showing the relationship between soft threshold power (1-20) and scale-free topology model fit (signed R²). **(D)** Mean connectivity plot displaying the relationship between soft threshold power and mean connectivity. **(E)** Gene dendrogram showing dynamic tree cutting results with minimum module size of 80 genes and deepSplit = 2. **(F)** Clustering dendrogram of module eigengenes with dissimilarity threshold of 0.25 for merging similar modules. WGCNA, Weighted Gene Co-expression Network Analysis; CD, Crohn’s disease; UC, ulcerative colitis.
