# Supplementary figures and images for: Multi-omics derivation of a core gene signature for predicting therapeutic response and characterizing immune dysregulation in inflammatory bowel disease
Source: Front Immunol. 2025 Jul 31;16:1611598. doi: 10.3389/fimmu.2025.1611598 (PMC12350116; doi:10.3389/fimmu.2025.1611598)

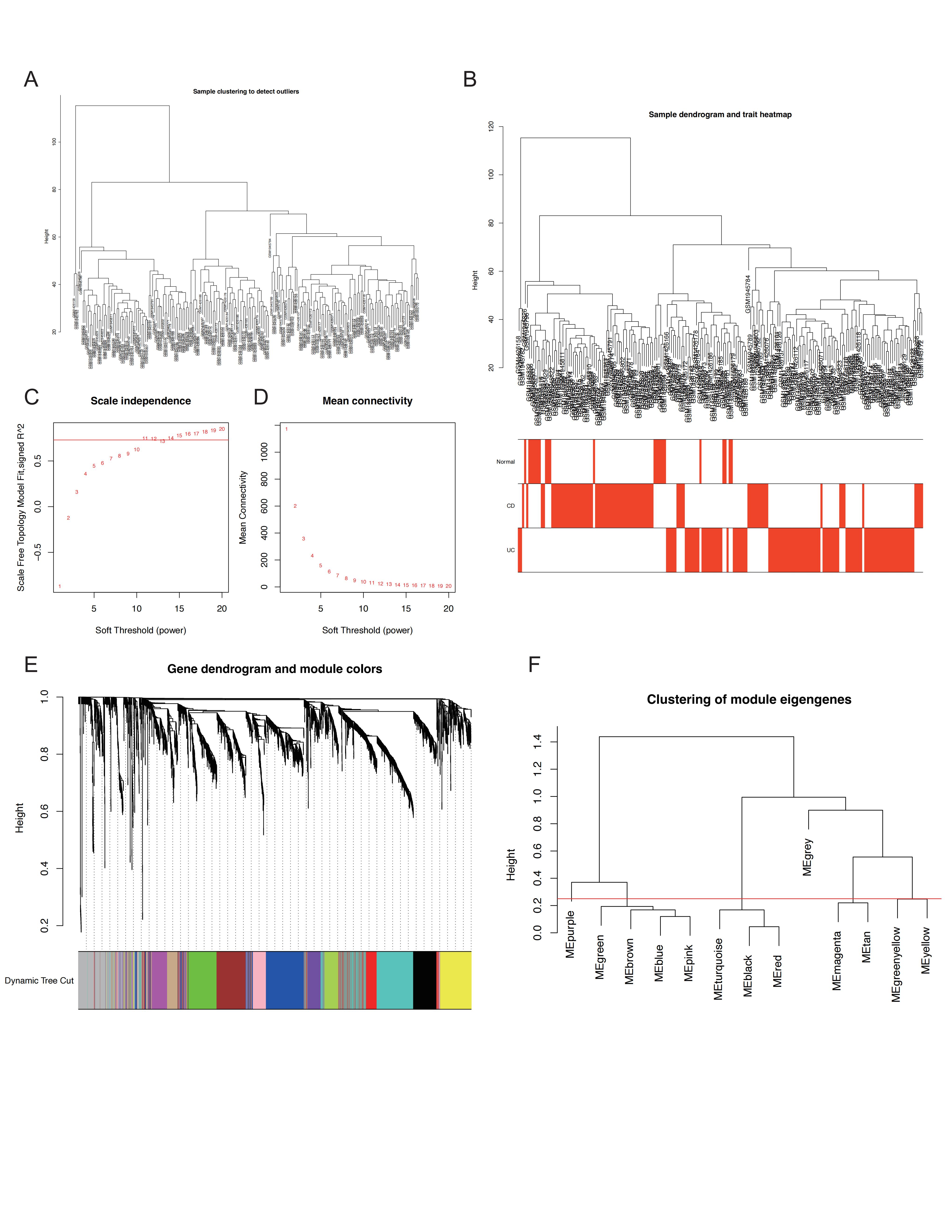

Supplement: Supplementary Figure 1 — WGCNA gene co-expression network construction and module identification. (A) Sample clustering dendrogram based on Euclidean distance and average linkage method, with a cutoff height of 12,000 applied to remove outlier samples. (B) Sample dendrogram and trait heatmap showing the relationship between sample clustering and clinical traits (Normal, CD, UC), with binary trait encoding (1 = present, 0 = absent). (C) Scale independence plot showing the relationship between soft threshold power (1-20) and scale-free topology model fit (signed R²). (D) Mean connectivity plot displaying the relationship between soft threshold power and mean connectivity. (E) Gene dendrogram showing dynamic tree cutting results with minimum module size of 80 genes and deepSplit = 2. (F) Clustering dendrogram of module eigengenes with dissimilarity threshold of 0.25 for merging similar modules. WGCNA, Weighted Gene Co-expression Network Analysis; CD, Crohn’s disease; UC, ulcerative colitis. [file Image1.jpeg]
